# Development of a Piezoelectric Vacuum Sensing Component for a Wide Pressure Range

**DOI:** 10.3390/s141122099

**Published:** 2014-11-21

**Authors:** Bing-Yu Wang, Fan-Chun Hsieh, Che-Yu Lin, Shao-En Chen, Fong-Zhi Chen, Chia-Che Wu

**Affiliations:** 1 Department of Mechanical Engineering, National Chung Hsing University, 250, Taichung 402, Taiwan; E-Mails: sunny_0419@hotmail.com (B.-Y.W.); fchsieh@itrc.narl.org.tw (C.-Y.L.); gear49761101@hotmail.com (S.-E.C.); 2 Instrument Technology Research Center, National Applied Research Laboratories, 20, R&D Rd. VI, Hsinchu Science Park, Hsinchu 300, Taiwan; E-Mails: fchsieh@itrc.narl.org.tw (F.-C.H.); chen@itrc.narl.org.tw (F.-Z.C.)

**Keywords:** vacuum pressure sensor, viscous forces, piezoelectric beam

## Abstract

In this study, we develop a clamped–clamped beam-type piezoelectric vacuum pressure sensing element. The clamped–clamped piezoelectric beam is composed of a PZT layer and a copper substrate. A pair of electrodes is set near each end. An input voltage is applied to a pair of electrodes to vibrate the piezoelectric beam, and the output voltage is measured at the other pair. Because the viscous forces on the piezoelectric beam vary at different air pressures, the vibration of the beam depends on the vacuum pressure. The developed pressure sensor can sense a wide range of pressure, from 6.5 × 10^−6^ to 760 Torr. The experimental results showed that the output voltage is inversely proportional to the gas damping ratio, and thus, the vacuum pressure was estimated from the output voltage.

## 1. Introduction

Recently, vacuum technology has occupied a key position in diverse fields of advanced science and technology such as surface science, thin film technology, space science, high-energy particle accelerators, microelectronics, and materials science. In addition, vacuum technology has an increasingly wide range of industrial production applications such as product packaging, vacuum casting, vacuum drying, chemical vapor deposition, evaporation, sputtering and dry etching. The vacuum pressure ranges from atmospheric pressure (760 Torr) to ultra-high vacuum pressure (10^−13^ Torr). For example, evaporation [1] must be conducted in vacuum pressures from 10^−7^ to 10^−5^ Torr to increase the evaporation rate and to maintain the purity and density of the film. Sputtering [2] must be conducted in vacuum pressure less than 10^−2^ Torr to have deposited thin films which are uniformly distributed on the substrate. The low pressure environment leads to reduction in the frequency of collision of ions with gas molecules, thus increasing the mean free path of the particles. In reactive ion etching [3], the etching operation is carried out at vacuum pressures below 10^−2^ Torr to increase directional etching. The low pressure environment leads to reduce the probability of collision of ions and neutral particles. In the electron cyclotron resonance plasma process [4], the operating pressure is controlled at below 10^−4^ Torr to achieve high density and uniformity. High density plasma [5] is operated in vacuum pressures from 10^−6^ to 10^−2^ Torr, which improves the etching rate and enhances the etching direction.

Vacuum covers a wide range of pressures. The mean free path of residual gas molecules is an important parameter that defines the vacuum state, which indicates the average distance travelled by molecules between collisions with each other. In initial stages of evacuation, *i.e.*, at low vacuum, the motion of gas is similar to gas flow. In high vacuum, *i.e.*, as the vacuum pressure is lowered below 10^−3^ Torr, the mean free path of residual gas molecules increases, and the motion of gas gradually becomes a molecular motion. For convenience, when discussing vacuum technology, we use different gas motions to distinguish between different vacuum states.

To achieve the vacuum condition, a mechanical pump is initially used to exhaust the gas at atmospheric pressure. This initial state of gas flow is called viscous flow [6] or continuous flow. In this state, the features of gas flow are the mutual collisions between each gas molecule, movement of each gas molecule limited by surrounding molecules, friction between the gas molecules, direction of gas flow, and gas molecules moving in the same direction. As the vacuum system continues the pumping action, the gas pressure continues to decline and the gas flow state becomes transition flow [7]. This gas flow state is very complicated which part of the gas flow maintains the viscous flow state but part of them converts into the molecular flow state. When the gas pressure of the vacuum system is reduced to a certain level, the gas flow state reaches the molecular flow range [8]. In this state, gas molecules have free random motion. The collisions are elastic and consistent with the conservation of kinetic energy and momentum conservation law. The probability of a gas molecule colliding with the chamber wall is greater than the probability of it colliding with another gas molecule. Regardless of how low the pressure is, the flow state maintains molecular flow once the flow conditions in the vacuum system reach the molecular flow range.

According to the operating principle, the vacuum gauges can be distinguished as absolute vacuum gauges and relative vacuum gauges. The operating principle of an absolute vacuum gauge involves the direct measurement of the forces on the unit area. The measurement principle of a relative vacuum gauge involves the use of the relationship between gas pressure and certain physical quantities. For example, the pressure can be obtained by thermal conduction [9–14]. Moreover, the pressure also can be measured by gas-molecule ionization technology indirectly [15–17].

Usually, we use different types of vacuum gauges to measure the vacuum pressure within the respective pressure ranges from 10^−7^ to 760 Torr. The vacuum gauges used in low vacuum (1–760 Torr) are based on an elastic element [18–22]. The vacuum gauges used in medium vacuum (10^−3^–1 Torr) are based on a thermal conduction element [10–14]. The vacuum gauges used in high vacuum (10^−7^–10^−3^ Torr) are based on a gas ionization element [15–17].

The abovementioned gauges include the diaphragm gauge [18–22], thermal conductivity vacuum gauge, ion vacuum gauge, and viscosity vacuum gauge [23]. The operating principle of the diaphragm gauge involves the measurement of the capacitance change caused by deformation of a film surface by pressure. The pressure value can be estimated based on the capacitance change. The operating principle of the thermal conductivity vacuum gauge involves the use of heat transfer from the objects heated by gas collision. Heat conduction is proportional to the frequency of gas molecule collisions with the objects. Therefore, the vacuum pressure is proportional to the heat conduction, enabling the estimation of pressure. The operating principle of the ion vacuum gauge involves the measurement of the number of molecules in the vacuum system to determine the pressure. The operating principle of the viscosity vacuum gauge involves the use of the viscosity characteristics of residual gas in the vacuum system to determine the pressure. The spinning rotor viscosity vacuum gauge [24,25] can be used to measure vacuum pressure ranges between 10^−1^ and 10^−7^ Torr. Gas viscosity caused by gas resistance is proportional to the degree of vacuum.

Based on existing literature, there are just few vacuum gauges that can measure the entire pressure range from 10^−7^ to 10^−2^ Torr. Current spinning rotor viscosity vacuum gauges can measure vacuum pressure ranges of 10^−7^–10^−1^ Torr. Due to the presence of several components such as the ball and control coil, spinning rotor viscosity vacuum gauges increases the volume, weight, and complexity of the system, thus limiting their applications. Mortet *et al.* [26] used a commercially available piezoelectric bimorph cantilever as pressure sensor which detected the change in the resonance frequencies due to the drag force of the surrounding gas. Sumali *et al.* [25] used a bulk piezoelectric transducer shook the whole chip on which an atomic force microscope probe mounted. A laser Doppler vibrometer (LDV) with a microscope measured the velocities at a point on the chip, and 42 points along the edges and tip of the cantilever. They stressed that the air damping is proportional to pressure in the rarefied regime. Wang *et al.* [27] developed a micro-cantilever beam deflected using electrostatic force. They measured the capacitance between two electrodes which were mounted around the proof mass of the trapezoidal micro-cantilever beam and a sensing electrode was placed on top of the proof mass with the deflection electrode mounted beneath to determine the free decay rate of the sensing beam with respect to deflection force and vacuum pressure. However, those devices still need additional actuators to drive the element and outside sensing devices to convert deflection of beams to electrical signals. Outside sensing devices make the measurement system complex.

In this study, we designed a self-actuated and self-sensing piezoelectric pressure sensor. The piezoelectric sensors are in the form of clamped–clamped beams. The sensor was designed using two piezoelectric elements: for self-actuating and self-sensing. Applying voltage to the PZT self-actuating element causes deformation of the clamped–clamped beams. At the other end of the beam, the PZT self-sensing element produces a voltage caused by the bending of the beam. Under different vacuum pressure values, the gas viscosity and the damping ratio of devices are different. This causes different swing amplitudes and resonant frequencies of the device and results to different output voltages and resonant frequencies from the sensing element. Thus, the vacuum pressure can be calibrated. The advantages of developed vacuum sensor are self-actuating and self-sensing without additional actuators and outside sensing elements. The wide range vacuum pressure from 6.5 × 10^−6^ to 760 Torr can be directly derived from piezoelectric output. Fabrication of developed vacuum sensors was easy because of simple structure.

This study has three specific goals. First, a piezoelectric pressure sensing element was developed that can be used to measure a wide range of vacuum pressure from 6.5 × 10^−6^ to 760 Torr. Second, the size of sensing element was 20 mm length, 5 mm width and 200 μm thick. Compared to commercial pressure sensors, the piezoelectric pressure sensors have the following advantages such as small size, low weight and simple instrumentation. Finally, the sensing elements were used to measure the pressure in nitrogen and argon to study the relationship between vacuum pressures and damping ratios of different gases.

## 2. Principle of Operation and Component Size

### 2.1. Principle of Operation

**Figure 1.**
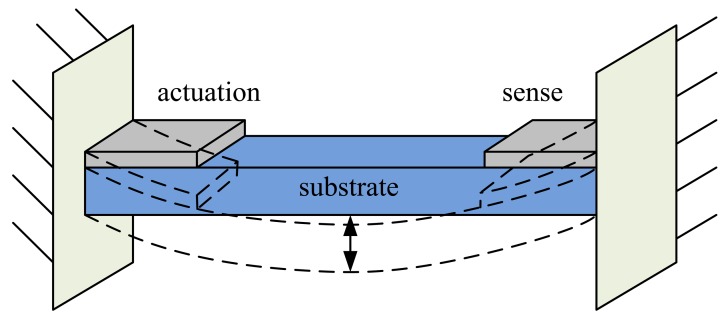
Schematic diagram of the piezoelectric vacuum pressure sensing component.

This study proposes the clamped–clamped beam-type piezoelectric vacuum pressure sensing element, a self-actuating and self-sensing microresonator, to detect the damping ratio of the gas, thus enabling the calculation of the pressure of vacuum system. The schematic diagram of the vacuum pressure sensing element is shown in Figure 1. The sensing element comprises a PZT layer, a substrate, and two pairs of electrodes. The electrodes are placed near both ends for piezoelectric actuation and sensing. When the sinusoidal voltage signal is applied to a pair of electrodes, due to the inverse piezoelectric effect, the clamped–clamped beam vibrates and resonates. Simultaneously, the other pair of electrodes captures the vibration energy and converts it to electric energy using the positive piezoelectric effect. Finally, we measured the output voltages which varied under different gases viscosity and vacuum pressures.

### 2.2. Choice of Component Materials

The piezoelectric sensor consists of a piezoelectric layer and a substrate. We choose PZT-5A as the piezoelectric layer and copper as the substrate. PZT-5A has a high piezoelectric constant and electromechanical coupling constant, and the energy consumption is small for conversion between mechanical energy and electrical energy. Copper has a low Young&s modulus and high electrical conductivity and can reduce the operating frequency. On the other hand, it can facilitate current conduction.

### 2.3. Component Size Design

The Euler–Bernoulli beam theory is the basis of assumptions to establish the mathematical model and is used to determine the size of the vacuum pressure sensing element. The force conservative equation is given as follows:

(1) $$\begin{array}{c} YI\frac{\partial^{4}w(x,t)}{\partial x^{4}}+c_{s}I\frac{\partial^{5}w(x,t)}{\partial x^{4}\partial t}+c_{a}\frac{\partial w(x,t)}{\partial t}+m\frac{\partial^{2}w(x,t)}{\partial t^{2}}+\alpha v_{in}\left(t\right)\left[\frac{d\delta (x)}{dx}-\frac{d\delta (x-x_{1})}{dx}\right]\\ +\alpha v_{out}\left(t\right)\left[\frac{d\delta (x-x_{2})}{dx}-\frac{d\delta (x-L)}{dx}\right]=0 \end{array}$$

where *YI* is the bending stiffness of the composite beam, indicating the resistance of the bending moment; *w(x,t)* is the cantilever deflection function, which is the neutral axis of the lateral displacement of each section (y-direction is positive); *c_s_I* is the equivalent damping term due to the viscosity of the combination cross section, where *c_s_* is the strain damping coefficient and *I* is the second moment of inertia for the combination of the cross section between the piezoelectric layer and substrate; *c_a_* is the air damping coefficient; *m* is the mass per unit length; and α is the piezoelectric coupling term. *v_in_(t)* and *v_out_(t)* are the input and output voltages, respectively. This cantilever piezoelectric sensing element contains two pairs of electrodes. The position of the input electrode ranges from *x* = *0* to *x* = *x_1_*, and that of the output electrode ranges from *x* = *x_2_* to *x* = *L*. Only the electric field is generated in the electrode coverage.

We assume that the operation of the piezoelectric sensor considers only the influence of resonance frequency (first mode). Therefore, the ratio of the output voltage to the input voltage from the mathematical model can be written as follows [28]:

(2) $$\left|\frac{v_{\text{out}}}{v_{\text{in}}}\right|=\frac{\left|\omega \tau_{c}\lambda_{1}x_{1}\right|}{\sqrt{{\left[-2\xi \tau_{c}\omega^{3}\right]}^{2}+{\left[2\xi \omega^{3}+\tau_{c}\lambda_{1}x_{2}\omega \right]}^{2}}}$$

where ω is the first resonance frequency of the cantilever, τ_c_ is the time constant of the output circuit, λ_1_ is the integrating factor, and ξ is the damping term in the modal coordinate functions. This damping term can be shown as Equation (3), and it combines the effect of air damping and structural damping:

(3) $$\xi =\frac{c_{s}I\omega }{2YI}+\frac{c_{a}}{2m\omega }$$

To reduce the sensor size in practical applications, the cantilever length and breadth are set as 20 and 5 mm, respectively, and the piezoelectric sheet thickness is set as 200 μm.

### 2.4. Electrode Design of Sensor

**Figure 2.**
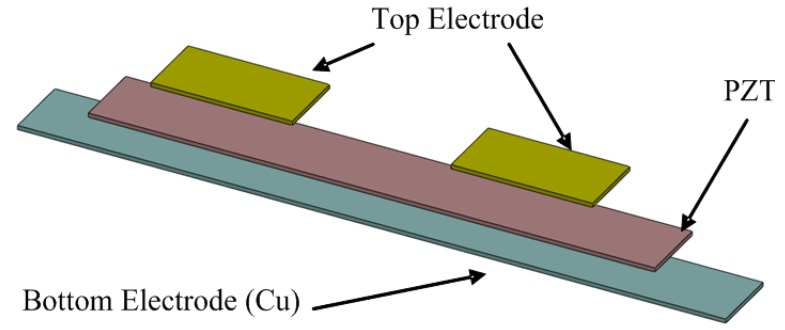
Top electrodes, PZT and bottom electrodes for piezoelectric vacuum sensors.

The two pairs of electrodes are distributed on the upper and lower surfaces of the piezoelectric material layer, which are placed on both ends of the cantilever. One pair of electrodes is placed beside the fixed boundary in the cantilever as an actuator to drive the cantilever and generate resonance. The other pair of electrodes is placed on the other side of the fixed boundary as a sensor to acquire the vibration energy and convert voltage signals, as shown in Figure 2. The electrode size is determined by the force conservative equation, thus leading to two designs. First, when the input electrode is close to the fixed end and has a length of 4.4 mm, the actuator can generate maximum power. Second, when the output electrode is close to the fixed end and has a shorter length, a larger open circuit output voltage is obtained. In our previous study, output voltages as high as twice the input voltages have been reported [29]. To obtain maximum power, we determine the length of the input and output electrodes to be 4.4 mm. The width and thickness of the electrodes were 5 mm and 10 μm, respectively. The upper electrode is fabricated using screen-printed silver.

### 2.5. Design and Production of Fixtures

**Figure 3.**
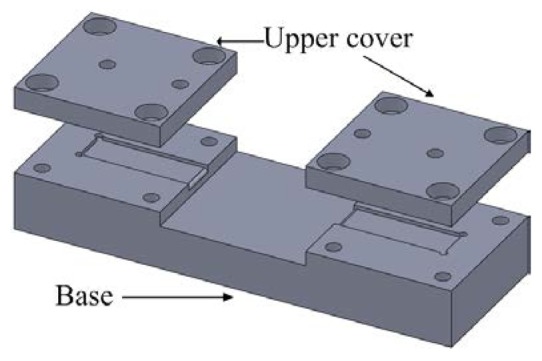
Schematic diagram of fixtures.

To allow the sensor to maintain the same boundary conditions during each measurement, we designed a fixture that can keep the cantilever beam fixed at both ends. The fixture contains two parts: upper cover and base. The upper cover has two holes for electrical wires. The clamped–clamped piezoelectric beam is placed in a trench in the base. Four M2 screws were used to fix the upper cover and the base which is shown in Figure 3. The fixture is made by transparent acrylic material which has advantages such as low density, high mechanical strength, good tensile and impact resistance, high transparency, low cost, and ease of machining.

## 3. Experimental Setup and Procedure

### Laboratory Equipment and Experimental Setup

The vacuum system consists of a stainless steel vacuum chamber, a mechanical pump, a turbo molecular pump, a gas flow controller and reference vacuum gauges. A mechanical pump (DOU 16B Balzers, Albuquerque, NM, USA) was first used to exhaust the gas at atmospheric pressure to achieve vacuum condition. When pressure was down to 10^−2^ Torr, a turbomolecular pump (Turbo VAC 450, Leybold, Cologne, Germany) was then used to obtain high vacuum (10^−2^ to 6.5 × 10^−6^ Torr). However, it might take more than 24 h to achieve 1 × 10^−6^ Torr using our pumping system. To reach certain pressure accurately, a gas flow controller was used to flow certain amount of nitrogen or argon into chamber. Two reference vacuum gauges, Pirani gauge and cold cathode gauge, were used. Pirani gauge is able to measure the pressure between 760 to 10^−3^ Torr and cold cathode gauge is capable to measure the pressure between 10^−2^ to 10^−9^ Torr.

**Figure 4.**
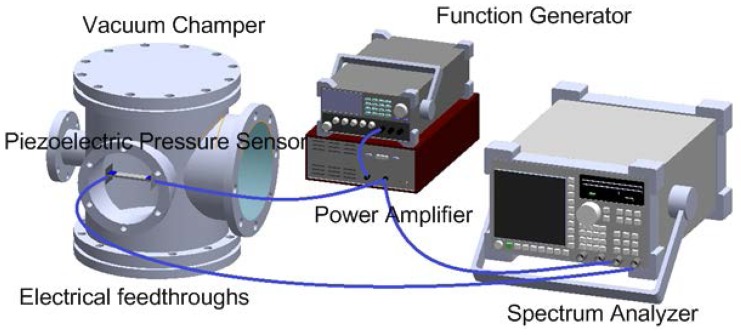
Experimental setup.

The experimental setup is shown in Figure 4. The clamped-clamped piezoelectric pressure sensor was fixed by the fixture and was placed in the vacuum chamber. Electrical feedthroughs were used to transfer electrical signals through the vacuum system wall. A sine wave was generated by a function generator (33220A, Agilent, Santa Rosa, CA 95403-1738, USA) and amplified by a power amplifier (PZD700, TREK, Lockport, NY, USA) to excite the piezoelectric beam at first resonance frequency. At the mean time, a spectrum analyzer (Agilent 35670A) was used to measure the frequency response of input and output voltages.

## 4. Results and Discussion

### 4.1. Frequency Response under Different Pressures

**Figure 5.**
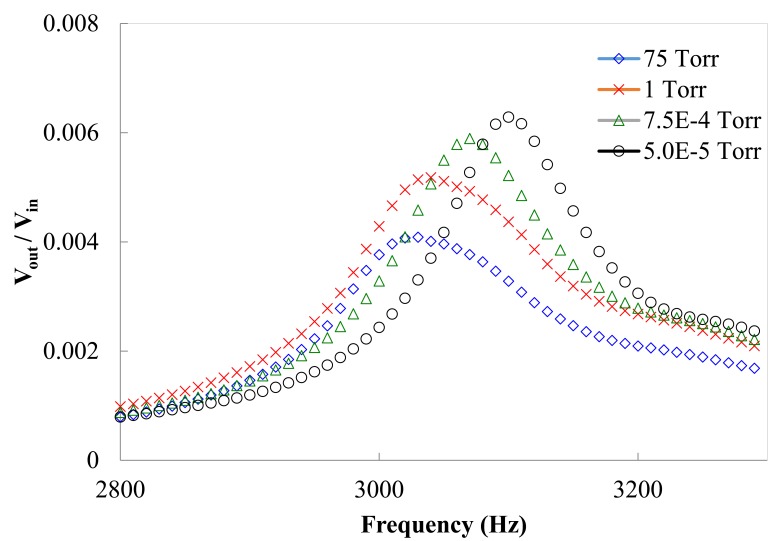
Frequency response of sensing components.

Frequency response functions were obtained by the following steps. The spectrum analyzer generated a swept-sine signal to drive a pair of electrodes through an amplifier. In the meantime, the other pair of electrodes generated electric output. Both the swept-sine signal and the output voltage were fed back to the spectrum analyzer to calculate the frequency response function. To maximum sensor output, the piezoelectric beams were excited at the first resonance frequencies under different pressures condition in the following experiments. The data from measured frequency response functions were processed to extract damping ratios using half power method. Figure 5 shows the frequency response functions of a clamped–clamped piezoelectric beam under different pressures when the residue gas in the vacuum chamber is nitrogen. The clamped-clamped piezoelectric beams have maximum output and input ratio when the beams were excited at resonance frequencies. The resonance frequencies of the piezoelectric beam under the vacuum pressures at 5 × 10^−6^, 7.5 × 10^−4^, 1 and 75 Torr were 3100, 3067, 3038 and 3030 Hz, respectively. When the pressures in the vacuum chamber decreased, the resonance frequencies and the resonance amplitudes of beam decreased because the damping coefficient of gas increased. The difference of first resonance frequencies of the vacuum sensor between 75 torr and 5 × 10^−6^ torr was just 2.25% (70 Hz) because of tiny damping variation. The resonance amplitude, output and input voltage ratio, were 0.0063, 0.0059, 0.0052 and 0.0032, respectively, when the pressures were 5 × 10^−6^, 7.5 × 10^−4^, 1 and 75 Torr.

### 4.2. Relationship between Pressure and Output Voltage

**Figure 6.**
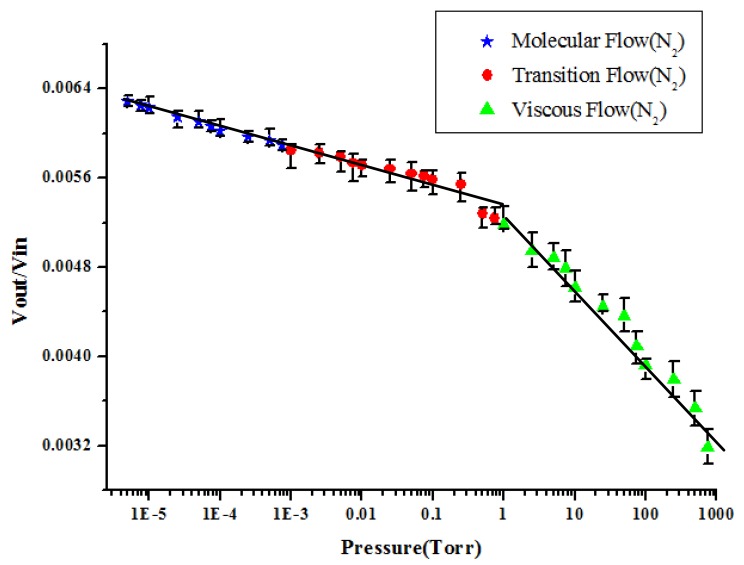
The resonance amplitudes (Vout/Vin) *versus* the vacuum pressures in the chambers when the residue gas is nitrogen.

Figure 6 showed the resonance amplitudes (Vout/Vin) *versus* the vacuum pressures in the chambers when the residue gas is nitrogen. Each operating frequency corresponding to the maximum amplitude values is applied in different vacuum pressure. In the experimental results, each pressure value corresponds to a piezoelectric output ratio. The vacuum pressure from 6.5 × 10^−6^ to 760 Torr can be directly derived from piezoelectric output ratio. Note that each experiments corresponding to different pressures were repeated 10 times. Finally, the average values of output voltage were reported. The data have been plotted on a log-log plot to show the extreme range of both the measured maximum amplitude and imposed air pressure. All the experimental results appear to have the same general trend, showing decreasing damping values with decreasing pressure. Clearly these experimental results support both the rarefied and the viscous theories. The pressure in the vacuum region is divided into three ranges for further analysis. Vacuum pressure below 10^−3^ Torr belonging to molecular flow is known as high vacuum; vacuum pressure in the range 10^−3^–1 Torr belonging to transition flow is known as medium vacuum; vacuum pressure greater than 1 Torr belonging to viscous flow is known as low vacuum. We use the linear regression method to deal with the results of each segment to obtain the best linear data. After processing, each line segment will be discussed. Pandey *et al.* [29] reported a paper to discuss effect of pressure on fluid damping in MEMS torsional resonators with flow ranging from continuum to molecular regime. Their results also indicated that the quality factors of devices varied in different flow regions.

The slopes of the voltage *vs*. pressure in the viscous flow region is y = −0.0002x + 0.0053 and R^2^ = 0.993. The slopes of the voltage *vs*. pressure in the transition flow region is y = −0.0003x + 0.0052 and R^2^ = 0.8913. The slopes of the voltage *vs*. pressure in the molecular flow region is y = −0.0007x + 0.0054 and R^2^ = 0.971. The slope of the voltage *vs*. pressure curve in the viscous flow region is greater than that in the transition flow region, while the slope in the transition flow region is greater than that in the molecular flow region. From this result, we infer that the gas viscosity force of viscous flow is greater than the other two flows. Also, the force due to change in gas viscosity for viscous flow is more obvious than that for transition flow and molecular flow. Therefore, the change in the piezoelectric output ratio is the most obvious in viscous flow. However, fitting in three regions may not be the best solution. It also looked that the data in the molecular flow and transition flow region could be fitted with one straight line instead of two. The slopes of the voltage *vs*. pressure from 5 × 10^−6^ to 1 torr is y = −0.00022x + 0.0053 and R^2^ = 0.924. The slopes of the voltage *vs*. pressure from 1 to 750 torr is y = −0.0007x + 0.0054 and R^2^ = 0.971.

### 4.3. Relationship between Pressure and Damping Ratio

**Figure 7.**
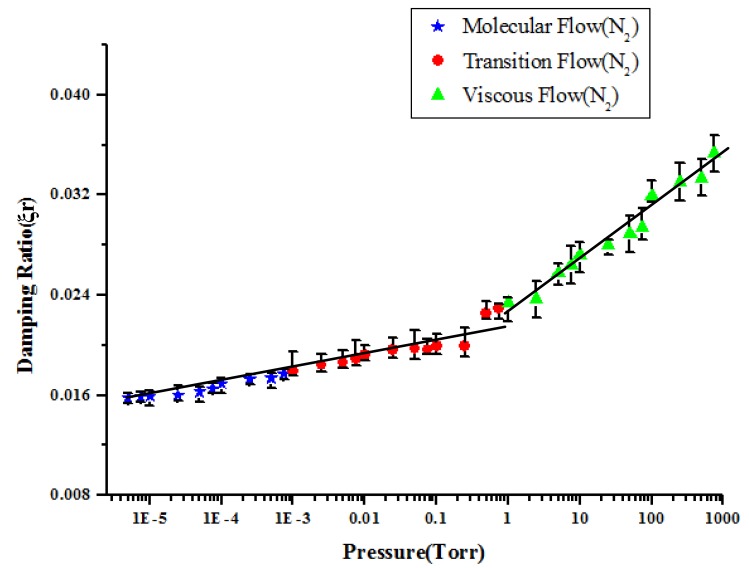
The damping ratios *versus* the vacuum pressures in the chamber when the residue gas is nitrogen.

The data from measured frequency response functions in the previous experiments were processed to extract damping ratios using half power method. After processing, we obtain the damping ratio corresponding to the respective pressure. Figure 7 showed that the damping ratios *versus* the vacuum pressures in the chamber when the residue gas is nitrogen. The slopes of the damping ratios *vs*. pressure in the viscous flow region is y = 0.0009x + 0.0206 and R^2^ = 0.957. The slopes of the damping ratios *vs*. pressure in the transition flow region is y = 0.0018x + 0.0226 and R^2^ = 0.8574. The slopes of the damping ratios *vs*. pressure in the molecular flow region is y = 0.0042x + 0.0225 and R^2^ = 0.9592. However, fitting in three regions may not be the best solution. It also looked that the data in the molecular flow and transition flow region could be fitted with one straight line instead of two. The slopes of the voltage *vs*. pressure from 5 × 10^−6^ to 1 torr is y = 0.0013x + 0.0216 and R^2^ = 0.916.

The measured damping ratio is the sum of structural damping and gas damping. Experimental results showed that the greater the quantity of residual gas in the vacuum chamber, the larger is the damping effect for the sensing element caused by the residual gas. Greater pressure indicates a larger number of gas molecules in the vacuum chamber. Therefore, there is a greater probability of collisions between gas molecules and piezoelectric beam; this situation increases the gas damping effect for the sensing element when the pressure increases. When the residual gas in the vacuum chamber was rarer, small damping effects for the sensing element were obtained experimentally.

### 4.4. Different Residue Gases—Nitrogen and Argon

**Figure 8.**
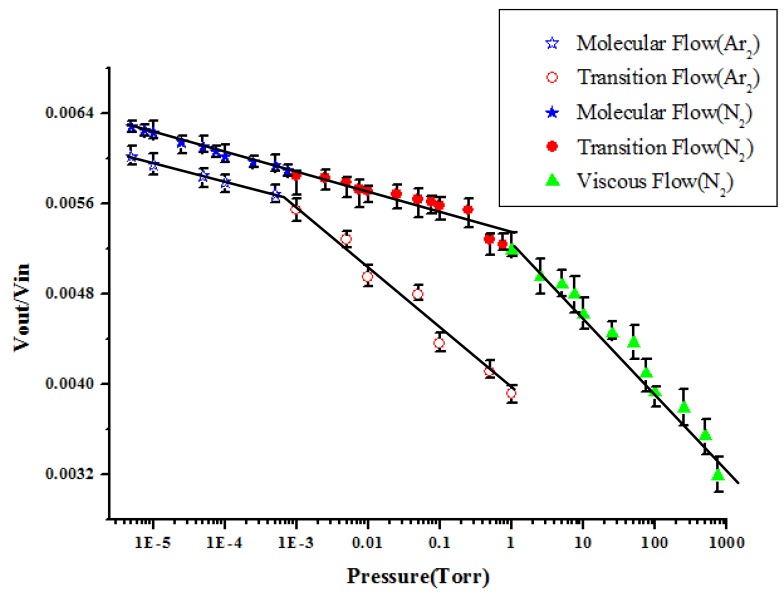
The resonance amplitudes (Vout/Vin) *versus* the vacuum pressures in the chambers when the residue gases are nitrogen and argon.

Figure 8 shows the resonance amplitudes (Vout/Vin) *versus* the vacuum pressures in the chambers when the residue gases are nitrogen and argon. However, the resonance amplitudes of the sensing element in the argon were just performed in the vacuum pressure from 6.5 × 10^−6^ to 1 Torr due to the limitation of the gas flow controller. We find that the output result has similar trends for both nitrogen and argon in the vacuum pressure from 6.5 × 10^−6^ to 1 Torr. The output value changes marginally. Under the same pressure, the output value of nitrogen is larger than that of argon. We can conclude that the vibration of the cantilever beam affected by the viscous force caused by argon is larger than that caused by nitrogen. The mass of argon (39.948 amu) is larger than that of nitrogen (28 amu). The resistance force of the molecular collision of argon is larger than that of nitrogen.

Each experimental corresponding to different pressures under Nitrogen and Argon was repeated 10 times and the average values of output voltage were reported. Experimental results were consistent if that boundary condition remained the same. Pressure measurement was taken after observing the steady state condition to prevent measurement errors. However, the duration of steady state condition varies for the flow in the continuous region to the flow in the molecular region. One of the samples was leaved in the vacuum chamber for 3 months and there were no significant difference even the sample was driven for a long time. However, there still need further research to study the consistency and stability of the system. There also still need further research to study the different gases to verify the consistency and stability of the system.

## 5. Conclusions

In this study, we developed a clamped–clamped beam-type piezoelectric vacuum pressure sensor. The sensor was designed using two piezoelectric elements: for actuating and sensing. Applying voltage to the PZT actuating element causes deformation of the cantilever. At the other end of the beam, the PZT sensing element produces a voltage caused by the bending of the beam. The piezoelectric pressure sensing element was developed that can be used to measure a wide range of vacuum pressure from 5 × 10^−6^ to 760 Torr. From low to high vacuum, the output and input voltage ratio (Vout/Vin) gradually increased with decrease in pressure. The relationship between vacuum pressure and damping ratio was obtained for pressure from 5 × 10^−6^ to 760 Torr. In high vacuum, the damping ratio is less than that in low vacuum. Finally, the sensing elements were used to measure the pressure in nitrogen and argon to study the relationship between vacuum pressures and damping ratios of different gases. Comparison of the output voltage ratios in argon and nitrogen showed that the damping ratios follow the same trend as the vacuum pressure. The damping ratio of argon is greater than that of nitrogen because the mass of argon is larger.
